# Impacts of nutrients and pesticides from small- and large-scale agriculture on the water quality of Lake Ziway, Ethiopia

**DOI:** 10.1007/s11356-016-6714-1

**Published:** 2016-04-28

**Authors:** Berhan M. Teklu, Amare Hailu, Daniel A. Wiegant, Bernice S. Scholten, Paul J. Van den Brink

**Affiliations:** 1 Department of Aquatic Ecology and Water Quality Management, Wageningen University, Wageningen University and Research Centre, P.O. Box 47, 6700 AA Wageningen, The Netherlands; 2 The College of Natural Sciences, University of Addis Ababa, Arat Kilo campus, Addis Ababa, Ethiopia; 3 Horn of Africa Regional Environment Centre and Network, Addis Ababa University, P.O. Box 80773 Addis Ababa, Ethiopia; 4 Alterra, Wageningen University and Research Centre, P.O. Box 47, 6700 AA Wageningen, The Netherlands

**Keywords:** Water quality, Monitoring, Physicochemical parameters, Pesticides, Risk assessment, Lake Ziway

## Abstract

The area around Lake Ziway in Ethiopia is going through a major agricultural transformation with both small-scale farmers and large horticultural companies using pesticides and fertilisers at an increased rate. To be able to understand how this influences the water quality of Lake Ziway, water quality data was gathered to study the dynamics of pesticide concentrations and physicochemical parameters for the years from 2009 to 2015. Results indicate that for some physicochemical parameters, including pH, potassium and iron, over 50 % of the values were above the maximum permissible limit of the Ethiopian standard for drinking water. The fungicide spiroxamine poses a high chronic risk when the water is used for drinking water, while the estimated intake of diazinon was approximately 50 % of the acceptable daily intake. Higher-tier risk assessment indicated that the fungicide spiroxamine poses a high acute risk to aquatic organisms, while possible acute risks were indicated for the insecticides deltamethrin and endosulfan. Longer-term monitoring needs to be established to show the water quality changes across time and space, and the current study can be used as a baseline measurement for further research in the area as well as an example for other surface water systems in Ethiopia and Africa.

**Electronic supplementary material:**

The online version of this article (doi:10.1007/s11356-016-6714-1) contains supplementary material, which is available to authorized users.

## Introduction

The current major agricultural transformation of Africa is increasing the anthropogenic sources of pollution, which are becoming a major concern for the environment (Pretty et al. 2011). One such example is the developments in the Central Rift Valley (CRV) of Ethiopia, where smallholder horticulture farmers and large-scale flower growing companies are located around Lake Ziway. The land use records of the area for the year 2006 indicate that the area of irrigated agriculture in the Lake Ziway catchment has increased up to 5000 ha since 1973. The total area of small-scale agriculture in the Meki River catchment (situated in the catchment of Lake Ziway) has been recorded to be of the order of 7300 ha, with a sharp increase especially after the year 1999 (Jansen et al. 2007). Farmers and companies in the area use pesticides and chemical fertilisers, which may affect the water quality of the lake and the surrounding surface waters through the release of some trace elements and residues from the agricultural fields into the surface waters (Jansen and Harmsen 2011). Although in most cases, both organic and inorganic pollutants are subjected to biodegradation, biotransformation and abiotic processes that reduce concentrations in the environment, this will not be the case for contaminants like persistent organic pesticides and heavy metals. These compounds may cause damage to aquatic organisms and humans because of their bioaccumulation in the food chain and high toxicity (Van Leeuwen and Vermeire 2007).

Hence, monitoring organic and inorganic pollutants in surface waters is essential to assess their risks to aquatic ecosystems and human health. The increased use of fertilisers by smallholders and enterprises, the increased volumes of (untreated) urban waste water and the reduced outflow from Lake Ziway to the Bulbula River can cause eutrophication, which can result in turbid water, algal blooms, fish mortality and poor quality of drinking water (Jansen and Harmsen 2011). In general, surface water is used as a source of drinking water in many rural parts of Ethiopia, so the Pesticide Risk Reduction Programme—Ethiopia (PRRP) has declared that priority should be given to the protection of Ethiopian surface waters as a source of drinking water for humans and as an important habitat for aquatic life (Teklu et al. 2015; Adriaanse et al. 2015).

In Ethiopia, this has only sporadically been done for specific water bodies for a longer period of time, mainly due to a lack of finances, skilled professionals and internationally certified laboratories. Moreover, the presence of very few nationally certified laboratories which can provide reliable results of residue analysis is hampering their publication as the societal sensitivity of the results implies that values have to be reliable.

Although a number of studies are being conducted in the Lake Ziway area catchment, most of them focussed on studying the hydro-geochemistry of waters around Lake Ziway by analysing samples from groundwater and surface waters using only a single measurement (Gashaw 1999), and on determining the low molecular mass (LMM) trace element species in the Ethiopian CRV lakes including Lake Ziway (Masresha et al. 2011). Others focus on the heavy metals and organochlorine pesticides present in fish species, sediment and water samples and the correlations between them (Nigussie et al. 2010; Dsikowitzky et al. 2012; Yohannes et al. 2014). These studies detected e.g. dichlorodiphenyltrichloroethanes (DDTs), hexachlorocyclohexanes (HCHs), chlordanes, heptachlors and heavy metals in organs of fish species in Lake Ziway.

To address the problems identified above, the present research aimed to obtain a better understanding of the water quality changes in Lake Ziway across time and space. To this end, a range of physicochemical parameters were repeatedly measured. In addition, the study monitored pesticide concentrations at several sampling points in Lake Ziway and compared the results with previously reported pesticide concentrations in the lake. To assess the potential risks of the measured pesticide concentrations to aquatic life, a tiered risk assessment, including species sensitivity distributions (SSD), was conducted. Overall, this study provides a comprehensive analysis of the water quality of Lake Ziway and its surrounding area, which so far has been described only to a limited extent.

The objectives of this study were (i) to determine the changes in the water quality of Lake Ziway across time and space; (ii) to evaluate whether the physicochemical and nutrient values of the water samples exceed international standards for drinking water and the aquatic community; (iii) to show the correlations between the various physicochemical parameters and their levels at the sampling locations, by performing a multivariate analysis; (iv) to examine the changes in the types and concentrations of pesticides recorded in the area and (v) to assess the risks posed by pesticides to aquatic organisms and to the use of the surface water as drinking water.

## Materials and methods

### Description of the study area

**Fig. 1:**
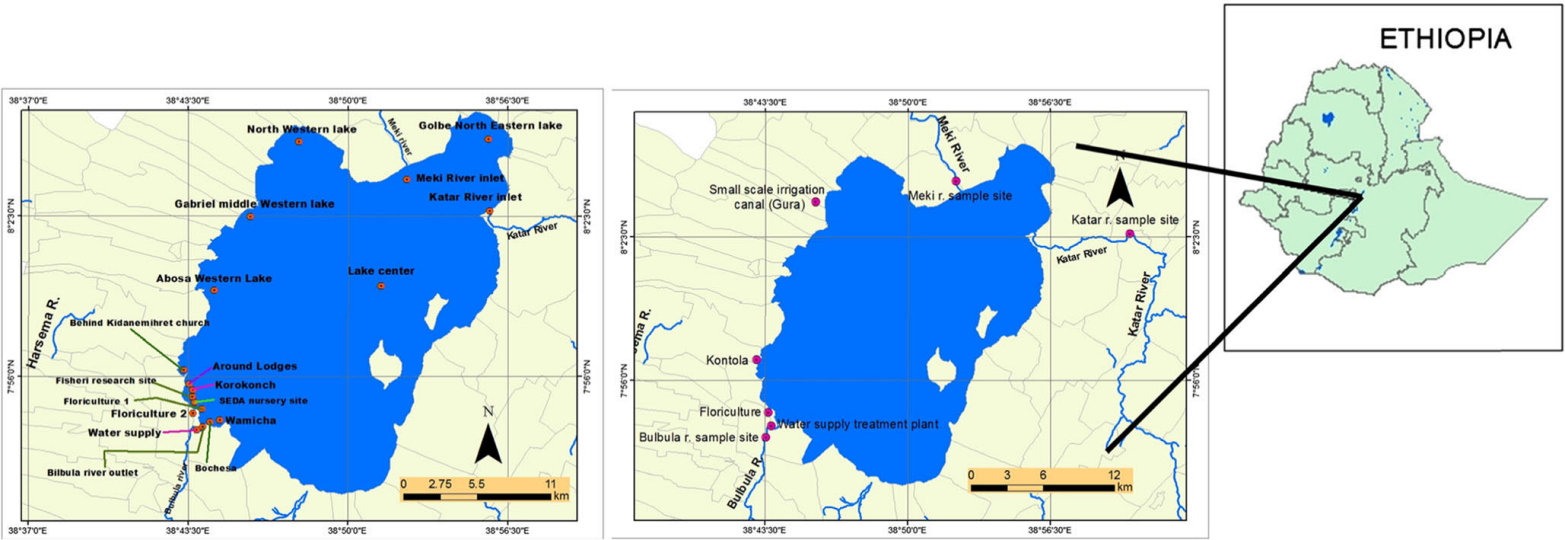
Physicochemical parameter and pesticide sampling points of Lake Ziway

The study was conducted at Lake Ziway, which is located in the CRV zone of Ethiopia (Fig. 1). It is situated in the East Showa zone of the Oromia region at about 160 km from Addis Ababa. Lake Ziway has an open water area of 434 km^2^, with an average depth of 4 m and an elevation of 1636 m above sea level. The Ziway Catchment is situated in between 7° 15ʹ N to 8° 30ʹ N latitude and 38° E to 39° 30ʹ E longitude, covering a total area of about 7300 km^2^ (Hughes and Hughes 1992). The lake has flat swampy margins on all sides except the south and south-west and is fed by many streams. The two main rivers, Meki and Ketar, flow into the lake, while one river, Bulbula, flows out of the lake (Jansen et al. 2007). Horticultural industries are situated between Lake Ziway and the main highway, at altitudes ranging between 1600 and 1700 m above sea level (Sahle and José 2013).

### Sample collection

Under close supervision and facilitation by the Horn of Africa Regional Environmental Centre and Networks (HoA-REC&N), the sample collection and analysis of physicochemical parameters by the nationally certified laboratory of Horticoop Ethiopia started by mid-2013 and continued until the beginning of 2015, once a month. During this period, samples were collected with the assistance of the local research institutions. At first, samples were collected from 13 locations, and this number increased to 18 between October 2014 and February 2015 (Fig. 1). The number of sampling locations was increased to better describe the spatial variability of the water quality parameters. In addition, seven samples were taken for pesticide residue analysis in August 2014 and March 2015, very close to the sampling locations used in previous research by Jansen and Harmsen (2011) (Fig. 1). Pesticide samples were shipped to Altic B.V. (NEN-EN-ISO/IEC 17025 accredited laboratory) in the Netherlands for pesticide residue analysis. Samples were preserved by adding 1 mL of acid, placed in a deepfreeze container and shipped in accordance with the US-EPA protocol for shipping and sample submission procedures for analytical services (US-EPA 2014). The commercial flower farms are expected to be characterised by a year-round nutrient load, whereas on the small-scale irrigated farms, high nutrient loads are expected between February and April and between June and October.

Grab sampling technique was used for all sampling activities (Forrest 2000). The containers were cleaned with distilled water and rinsed two to three times with water at the sampling point before the sample was taken. The collected water was thoroughly mixed and checked to see if all organic matter such as leaves, rags, twigs and other floating materials had been removed. All collected 1-L samples were placed in a labelled sample container and stored in a refrigerator at 4 °C.

### Physiochemical properties and pesticide residues

At Horticoop Ethiopia, the nutrients N, Ca, Mg, Na, S, P, Si, Cu, B, Fe, K, Mn, Mo and Zn concentrations were measured simultaneously using an optimised and frequently calibrated device for inductively coupled plasma analysis. Chloride was analysed by titration against AgNO_3_ solution and by weighing the precipitated chloride, bicarbonate was analysed by titration of the sample with HCl solution, ammonium-nitrogen and nitrate-nitrogen (NH4 + N, NO_3_-N) were analysed by spectrophotometric analysis (Janway 63 UK 2009) and pH and electrical conductivity (EC) were measured by direct reading in the water sample using an electrode pH meter (pH06, Holland 2006) and an EC Meter (EC 93 Holland 2010). All samples were filtered through a Whitman No. 42 filter paper before further analysis was done. Samples sent to the Dutch lab (Altic B.V.) were analysed for more than 300 pesticide residues. Samples were extracted using dichloromethane and petroleum ether, while analysis was performed using standard GC-ECD, GC-MS/MS and LC-MS/MS techniques (Chauhan et al. 2014; Adeyemi et al. 2011; Pitt 2009).

### Data analysis

The Ethiopian maximum permissible limits (MPL) as listed by the Ministry of Health (MoH 2011) and the World Health Organisation (WHO 2010) were used to identify those physicochemical parameters which exceeded the MPL. A multivariate analysis was done to assess the variation in the values of the physicochemical parameters across time and space (Van den Brink et al. 2003a). To this end, a redundancy analysis (RDA) was performed using the measured physicochemical parameters as response variables, while using the sampling locations and sampling dates as explanatory variables. The significance of the explanatory variables was evaluated by a Monte Carlo permutation test following the RDA. More information on the interpretation of RDA-derived biplots can be found in Van den Brink et al. (2003a).

Pesticide concentrations were available for four sampling dates. Data from two sampling dates in 2009 and 2010 was taken from Jansen and Harmsen (2011), while the 2014 and 2015 data was collected for the present study. Pesticide residues detected in each sampling period were first used to determine the acute exposure toxicity ratio (ETR) using predicted no-effect concentrations (PNECs) based on acute values for fish, daphnia and algae for each detected pesticide, as described in Teklu et al. (2015) and Wipfler et al. (2014), and are given below in Eqs. 1–3). The ETR was computed by dividing the measured concentration by the PNEC, using the maximum value of concentrations and the minimum value of the three PNECs for risk (ETR) calculation. SSDs and HC5 (hazardous concentration protective of 95 % of the population) concentrations were determined using acute toxicity data for additional species from the US-EPA database (www.epa.gov/ecotox) and the ETX 2.0. software (Van Vlaardingen et al. 2003). In the second-tier risk assessment, the HC5 values were used as PNEC values, following the recommendations by Maltby et al. (2005, 2009) and Van den Brink et al. (2006) for short-term exposure. For insecticides, the 1–7-day EC50 arthropod data was included in the SSD. The SSDs of the fungicides included 2–21-day EC50 data for vertebrates, 1–7-day EC50 data for invertebrates, 2–28-day EC50 data for macrophytes and 1–7-day EC50 data for algae (Maltby et al. 2005, 2009; Van den Brink et al. 2006). In case more than one value was available for a species, the geometric mean of the available values was used. Human health risk assessment was carried out for pesticides exceeding the EU’s threshold level for drinking waters of 0.1 μg/L (Dolan et al. 2013). Risks to humans was determined using the highest detected concentrations. Chronic and acute health risks were calculated using acceptable daily intake (ADI) and acute reference dose (ARfD) values, respectively. Estimated short-term intake (ESTI) and the internationally estimated daily intake (IEDI) values were determined to indicate acute and chronic risks to humans from drinking surface water as a source of drinking water. Background calculation formulas are given in Teklu et al. (2015), Wipfler et al. (2014) and Adriaanse et al. (2015). Risks were categorised as ETR <1: negligible to low risk; 1 < ETR < 10: possible risk and ETR >10: high risk to aquatic organisms and as ESTI or IEDI >100 %: high risk and ESTI or IEDI <100 %: low to negligible risk for humans assuming surface water is used as a source of drinking water.

PNEC_acute_ is the lowest of the acute PNEC for fish, daphnia and algae (μg/L), where

1 $$ \mathrm{PNE}{\mathrm{C}}_{\mathrm{fish}\ \mathrm{acute}}={0.01}^{\ast }\ \mathrm{LC}5{0}_{\mathrm{fish}}\left(\upmu \mathrm{g}/\mathrm{L}\right) $$

2 $$ \mathrm{PNE}{\mathrm{C}}_{\mathrm{daphnia}\ \mathrm{acute}}={0.01}^{\ast }\ \mathrm{EC}5{0}_{\mathrm{Daphnia}}\left(\upmu \mathrm{g}/\mathrm{L}\right) $$

3 $$ \mathrm{PNE}{\mathrm{C}}_{\mathrm{algae}\ \mathrm{acute}}={0.1}^{\ast }\ \mathrm{EC}5{0}_{\mathrm{algae}}\left(\upmu \mathrm{g}/\mathrm{L}\right) $$

with PNEC_fish-acute_ = predicted no acute effect concentration for fish (μg/L); LC50_fish_ = concentration that kills 50 % of the test organisms, fish; EC50_Daphnia_ = concentration that affects 50 % of the test organisms, daphnia, and EC50 algae = concentration that inhibits the algae growth by 50 %; the values 0.1 and 0.01 are the safety factors used for algae and fish and daphnia, respectively.

## Results and discussion

### Physicochemical parameters

**Fig. 2:**
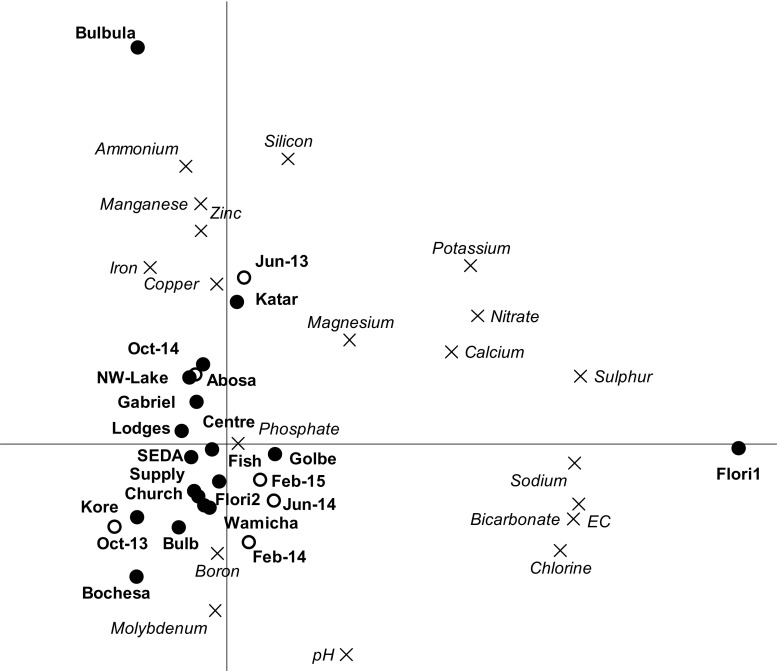
RDA biplot showing the correlations between sampling date and site and the physicochemical parameters. Sampling date and site explained a significant part of the variation in physicochemical parameter value (44 %; *p =* 0.01) levels. Of this variation, 34 % is displayed on the *horizontal axis* and another 20 % on the *vertical axis*. See Fig. 1 for locations of sampling points

Table 1 summarises the maximum, minimum and average values of all measured physicochemical parameters. The column headed ‘Percentage of values above the MPL’ shows that more than 50 % of collected samples exceeded the MPL for the parameters pH (59 %), potassium (87 %) and iron 100 %), while less than 10 % of the observed values exceeded the MPL for the parameters EC, ammonium, nitrate and boron (7 %), sodium (6 %) and manganese (1 %). All observed values for calcium, magnesium and chloride were below the MPL. The amounts of phosphorus, zinc, copper and molybdenum found in this study were either zero or too low to be detected (<0.01 mg/L) so they can be considered to be below the MPL even though no specific value was available. They can therefore be considered as posing no risk to human health (Table 1). The RDA biplot (Fig. 2) displays the temporal and spatial variation of the nutrients and physicochemical parameters. It appears that the sampling point at the Bulbula River had a high correlation with the vertical axis, which explains 20 % of the variation among the physicochemical parameters, while the horizontal axis, where floriculture 1, the main liquid waste outlet point of the floriculture area, had a high correlation, explains 34 % (*p* < 0.01). These results are in line with recorded changes in land use, which show that the numbers of both commercial (floriculture, horticulture and viticulture) and irrigated small-scale farming activities are increasing rapidly in the Lake Ziway catchment and its surrounding area. The accompanying contamination of the lake and other water bodies as a result of the use of intensive agriculture inputs like fertilisers may pose risks to human health when the water is used as drinking water (Jansen et al. 2007).

**Table 1:** Results for physicochemical and nutrient parameters in this study

Nutrient/physicochemical parameter	Number of observations	Min.	Mean	Max.	Ethiopian (WHO) MPL	% Of values above MPL
pH (–)	87	7.6	8.5	9.0	6.5–8.5	59
EC (μS/cm)	87	140	474	1740	1000	7
Ammonium (NH_4_^+^; mg/L)	87	0.01	0.64	3.1	1.5	7
Nitrate( NO_3_^−^; mg/L)	87	0.06	26	296	50	7
Phosphorus (mg/L)	87	<0.01	<0.01	<0.01	NA	<MPL
Potassium (mg/L)	87	0.33	14	53	1.5	87
Calcium (mg/L)	87	0.43	18	39	75	<MPL
Magnesium (mg/L)	87	0.38	8.1	29	50	<MPL
Sodium (mg/L)	87	3.2	72	337	200	6
Sulphur (mg/L)	87	0.09	3.8	20	NA	NA
Chloride (mg/L)	87	0.35	15	38	250	<MPL
Bicarbonate (mg/L)	87	3.9	257	704	NA	NA
Silicon (mg/L)	87	0.53	18	81	NA	NA
Iron (mg/L)	87	0.06	2. 6	29	0.3	100
Manganese (mg/L)	87	<0.01	0.033	0.90	0.5	1
Zinc (mg/L)	87	<0.01	<0.01	<0.01	5	<MPL
Boron (mg/L)	87	<0.01	0.22	5.7	0.3	7
Copper (mg/L)	87	<0.01	<0.01	<0.01	2	<MPL
Molybdenum (mg/L)	87	<0.01	<0.01	<0.01	NA	<MPL

*Min* minimum value, *Mean* mean of all observations, *Max* maximum value, *Ethiopian (WHO) MPL* Ethiopian maximum permissible limits for drinking water as listed in MoH (2011) and WHO (2010), which are similar

Previous research results on the Lake Ziway and CRV area indicated that the percentages of measurements exceeding the MPL where 13 % for pH and 1 % for EC, while 28 % of the iron levels were reported to exceed the MPL (Reimann et al. 2003). The single measurement values of pH (8.5) and EC (463 μS/cm) reported in Gashaw (1999) are comparable to the mean results obtained in this study of 8.5 and 474 μS/cm, respectively. Further comparison of these values with a study done in a Kenyan Lake shows that the pH and EC values there were within the WHO (Ethiopian) MPL for pH (7.5–8.5) and EC (566–601 μS/cm) (Ouma and Mwamburi 2014). In the present study, higher EC values were found for sampling points near the floriculture area (Fig 2). In an aquatic environment, EC is an important and simple indicator to characterise the pollution status of surface waters, as a sudden increase in conductivity can indicate the presence of more dissolved ions, which may have an impact on aquatic life and water quality.

### Pesticide detection

**Fig. 3:**
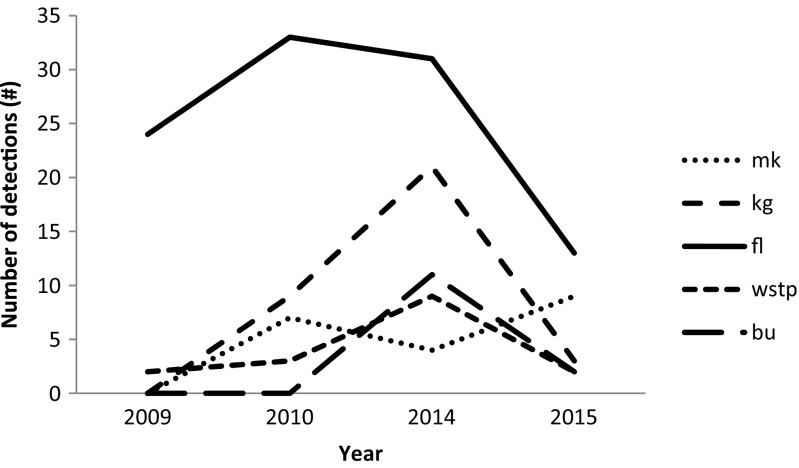
Numbers of detections over the years of sampling at different sampling locations. Note: *mk* Meki and Ketar rivers, *kg* Kontola and Gura, *fl* floriculture, *wstp* water supply treatment plant, *bu* Bulbula. Note that values for 2009 and 2010 are taken from Jansen and Harmsen (2011)

The pesticide residue analysis, including the results from the earlier study by Jansen and Harmsen (2011), indicated that overall detections of pesticides showed an increasing trend across the sampling years, but decreased in the final year of our study, with lower amounts of pesticides detected in 2015 (Fig. 3). Relatively few different types of pesticides were detected in the Meki and Ketar rivers, Bulula and water supply treatment plant areas, while the floriculture area had the highest total number of detections of pesticides, followed by Kontola and Gura (Fig. 3). High detection results are ascribed to the growing trend towards land use change on the small- and large-scale farms in the vicinity (Jansen et al. 2007). The environmental impact from the expansion of large-scale flower farms in Ethiopia in general and around Lake Ziway area in particular is of major concern for many environmentalists (Getu 2009). The trend towards increasing use of pesticides in the small-scale irrigated area as the sole pest management technique in the CRV also caused the high residue detections in the Kontola and Gura areas until the year 2014. This was shown by a survey conducted by the Pesticide Action Network—United Kingdom (PAN-UK) on the pesticide use and management by small-scale farmers in the CRV of Ethiopia. The survey found that 97 % of respondents reported using pesticides once or twice a year, and about 91 % of the farmers who were interviewed prepared their pesticides close to water sources used by local people for drinking, cooking and other household purposes. Sixty-one percent of the respondents washed their pesticide sprayers and other equipment on the farm field (PAN-UK 2006). The decreasing number of detections in the floriculture area in the years 2014 and 2015 might be associated with the recent innovations in liquid waste management. Flower farms are increasingly adopting mechanisms to minimise the environmental impacts of pesticides in the surrounding surface water systems, e.g. by constructing soakaway pits and wetlands to meet the standards of fair trade as a sustainable certified flower producer (Teklu et al. 2015).

Detection of DDT was reported by the earlier Jansen and Harmsen (2011) study, though no DDT was detected in the samples taken by HoA-REC&N. Small amounts of other pesticides found in the earlier study may be a result of the dilution and decay of pesticides in oxygen-rich river waters (Jansen and Harmsen 2011).

### Acute risks of pesticides to aquatic organisms

The results of the first tier-risk assessment (Table 2) show that chlofentezin, sulphur, spiroxamine and methomyl pose a high acute risk to aquatic organisms, with highest ETR values of 13, 11, 190 and 36, respectively. The table shows that the first-tier risk assessment based on PNEC values indicates higher risk values than the ETR based on HC5 values. This is consistent with the principles of the tiered approach, i.e. greater realism and less conservatism at higher tiers (Brock et al. 2006; Wheeler et al. 2002). There are, however, two exceptions, viz. spiroxamine and endosulfan. The lower and upper limits of HC5 and HC50 and the number of data points on which the SSD was based are provided as Supplementary Information (Annex 1).

**Table 2:** Risks to aquatic organisms calculated from 1st tier PNEC values (μg/L) and 2nd tier SSD HC5 values (μg/L)

Pesticides	Location	Year of highest detection	Maximum concentration (μg/L) (*n* = 4)	PNEC 1st tier	ETR 1st tier	SSD HC5	ETR SSD
Sulphur	mk	2010	7.0	0.63	11	690	0.01
Endosulfan	mk	2014	0.14	0.10	1.4	0.05	2.9
Diazinon	kg	2014	0.28	0.10	2.8	0.51	0.55
Dodemorph	kg	2014	32	22	1.5	NA	NA
Lufenuron	kg	2014	0.080	0.01	6.2	NA	NA
Spiroxamine	kg	2014	57	0.30	190	0.18	317
Sulphur	kg	2010	3.0	0.63	4.8	690	<0.01
Teflubenzuron	kg	2014	0.03	0.03	1.1	0.47	0.07
Methomyl	fl	2009	2.7	0.08	36	11	0.26
Spiroxamine	fl	2009	4.0	0.30	13	0.18	22
Teflubenzuron	fl	2014	0.05	0.03	1.8	0.47	0.10
Trifloxystrobin	fl	2010	0.34	0.15	2.3	1.3	0.27
Carbendazim	fl	2009	9.1	1.5	6.1	19	0.48
Chlofentezin	fl	2010	0.10	0.01	13	NA	NA
Deltamethrin	wtp	2014	0.01	<0.01	3.8	<0.01	4.1
Diazinon	wtp	2014	0.41	0.10	4.1	0.51	0.80
Endosulfan	wtp	2014	0.10	0.10	1.0	0.05	2.1
Lufenuron	wtp	2014	0.02	0.01	1.4	NA	NA
Pyraclostrobin	wtp	2015	0.06	0.06	1.0	0.35	0.18
Sulphur	wtp	2010	10	0.63	16	690	0.01
Teflubenzuron	wtp	2014	0.08	0.03	2.9	0.47	0.17
Spiroxamine	bu	2014	6.9	0.30	23	0.18	38

Only pesticide–location combinations with a 1st tier ETR >1 are included in the table. ETR <1: negligible/low risk; ETR >1: possible risk; ETR >10: high risk

*mk* meki and ketar rivers *kg* kontola and gura, *fl* floriculture, *wtp* water supply treatment plant, *bu* bulbula, *NA* not available

Model-based risk assessment of pesticides in surface waters in Ethiopia indicates that endosulfan is predicted to pose high risks in lowland ponds in Ethiopia (ETR = 32, with a PEC of 0.63 μg/L) while deltamethrin is predicted to pose only a possible risk (ETR = 2.5, with a PEC of 0.0066 μg/L) (Teklu et al. 2015). Although there are many explanations for this difference in concentrations, the most obvious one is the fact that the predicted values represent the 90th percentile of the 33-year predicted environmental concentration (PEC) values just after application (Adriaanse et al. 2015; Teklu et al. 2015), while a measured value is just the outcome of an ordinary measurement not necessarily taken close a pesticide application in space and time. In our study, the highest risk quotient for aquatic organisms for endosulfan was ETR 2.9, with the highest measured concentration (0.14 μg/L) found in the area of the Meki and Ketar rivers, while the highest measured concentration of deltamethrin, with an ETR of 4.1, was found at the water supply treatment plant, with a measured concentration of 0.01 μg/L; both are in the possible risk category (Table 2).

The overall acute risk assessment for aquatic organisms based on HC5 values indicates high risk values for the locations Kontola and Gura (spiroxamine 317), floriculture (spiroxamine 22) and Bulbula (spiroxamine 38), based on a stricter risk category (ETR >10) (Table 2). Spiroxamine is a fungicide which has been authorised for the control of the powdery mildew infestation that prevails in flowers in Ethiopia and which is also used in most European countries. Spiroxamine has a short half-life in the water phase (0.8 days). Algae are most sensitive to spiroxamine, followed by invertebrates and fish (University of Hertfordshire 2013; PHRD 2015). Though no data is available to compare the amounts and frequency of use of this fungicide in small- and large-scale farms, it is probable that a high risk is associated with the frequent use of this pesticide for fungal disease control in the area (Table 2).

### Human risk assessment

The human risk assessment was performed for values exceeding the European 0.1 μg/L standard (Table 3) and indicated that no acute risk to humans is present when the surface water is used as a source of drinking water (Table 4). This result is in line with Teklu et al. (2015), in which seven registered pesticides were evaluated using model-based risk assessment, and all were found to pose low or negligible acute risks to human health. The fungicide spiroxamine poses a high chronic risk to humans while the insecticide diazinon has the second highest IEDI value (47 %), followed by methomyl (36 %) and metalaxyl (25 %) (Table 4). It is unknown whether pesticides with values above 10 % will pose a risk when exposure through other food sources than water is also taken into account. Further investigation is required to assess the presence of high risk in combination with other food sources for the Ethiopian case, in order to get an overall risk estimation for these pesticides (Van den Brink et al. 2003b; Teklu et al. 2015).

**Table 3:** Measured pesticides concentrations above 0.1 μg/L (data from this paper and from Jansen and Harmsen 2011)

Location	Number > EU 0.1 μg/L	Pesticide with the highest score	Pesticide with the lowest score	Max. value (μg/L)	Min. value (μg/L)
Meki and Ketar	7	Metalaxyl	Endosulfan	59	0.095
Kontola and Gura	9	Spiroxamine	Cyprodinil	57	0.11
Floriculture	33	Boscalid	Clofentezine	13	0.17
Water supply treatment plant	4	Sulphur	Endosulfan	10	0.061
Bulbula	4	Spiroxamine	Buprofezin	6.9	0.081

**Table 4:** Acute and chronic human risk assessment results

Location	Compound	PEC (μg/L)	ARfD (mg/kg bw/day)	ADI (mg/kg bw/day)	ESTI (%)	IEDI (%)
Meki and Ketar	Metalaxyl	59	0.5	0.08	1.2	25
Kontola and Gura	Diazinon	0.28	0.025	0.0002	0.11	47
	Dodemorph	32	0.33	0.082	0.97	13
	Spiroxamine	57	0.1	0.015	5.7	127
Floriculture	Boscalid	13	NA	0.04	NA	11
	Methomyl	2.7	0.0025	0.0025	11	36
	Carbendazim	9.1	0.02	0.02	4.6	15
Bulbula	Spiroxamine	6.9	0.1	0.015	0.69	15

Data is presented only for pesticide–location combinations with an IEDI values above 10 %

Examination of the number of detections indicates that the locations Kontola and Gura and floriculture had the highest numbers of pesticide detections (Fig. 3). The fungicides metalaxyl and spiroxamine reported the highest concentrations, 59 and 57 μg/L, respectively (Table 3). Metalaxyl is a registered fungicide sold as Folio Gold 537.5 SC for the control of botrytis and downy mildew in flowers in Ethiopia and in some parts of European countries as well (University of Hertfordshire, 2013; PHRD 2015).

## Conclusion and recommendations

Comparison of our findings with local and international standards indicates that some physicochemical parameters and nutrients exceeded the local and international standards in more than 50 % of the cases. The results of our multivariate analysis indicate that the Bulbula outlet and the floriculture outlet are important sources of the variation in some of the physicochemical parameters in this study. These are places reported in an earlier land use study as undergoing major land use changes as regards commercial and small-scale irrigated agriculture.

The numbers of pesticide detections in this study and the amounts detected show an decreasing trend relative to the findings reported by Jansen and Harmsen (2011). This result is encouraging for further liquid waste management activities in the small- and large-scale farming activities in the area. Moreover, only one pesticide was found to pose a high acute risk to aquatic organisms (spiroxamine), while endosulfan and deltamethrin were found to pose a possible risk. All the pesticides pose no or negligible acute risk to humans if surface water is used as a source of drinking water while high chronic risk is expected from spiroxamine.

Some pesticide sampling points are located outside Lake Ziway, but these points are in direct water contact with the lake, so they can contaminate the sampling points at the edge of the lake. Even though it is believed that the overall dilution factor of the lake will reduce the concentration within the lake, maximum care should be taken in controlling liquid farm wastes on both the small- and large-scale farms. This requires constant interventions in liquid waste management in these areas. Commercial horticultural farms that produce vegetables and cut flowers need to develop or improve the use of mechanical and biological pest management techniques to reduce the use of synthetic chemicals. Together with the relevant authorities, they should extend their best practices for safe ecological pest management and their successful waste water control and treatment experiences to the surrounding small-scale horticulture farms, while waste coming from the commercial farms must also be regularly assessed.

Further investigations should focus on refining the results of this study and further investigating the dynamics of pesticides and physicochemical parameters and their exceedance of the MPL of the Ethiopian standard or the exceedance of the EU 0.1 μg/L standard in the case of pesticides. The chronic risk to humans and aquatic organisms also needs to be assessed, as some of the pesticides may be used regularly. Future studies should also consider studying the ecological risk due to mixtures of pesticides. Planning a continuous monitoring campaign together with all stakeholders concerned, including commercial and small-scale farmers and governmental and non-governmental organisations, is important to ensure sustainable water quality in the Ziway Lake catchment.

## Electronic supplementary material

Below is the link to the electronic supplementary material.

ESM 1 (DOCX 58 kb)
