# Supplementary material for: Impacts of nutrients and pesticides from small- and large-scale agriculture on the water quality of Lake Ziway, Ethiopia
Source: Environ Sci Pollut Res Int. 2016 Apr 28;25(14):13207–16. doi: 10.1007/s11356-016-6714-1 (PMC5978843; doi:10.1007/s11356-016-6714-1)
Supplement: Supplementary file 1 — (DOCX 58 kb) [file 11356_2016_6714_MOESM1_ESM.docx]

***Supplementary material to the paper published in Environmental Science and Pollution Research:***

**Impacts of nutrients and pesticides from small and large scale agriculture on the water quality and ecology of the Lake Ziway catchment, Ethiopia**

*Berhan M. Teklu^1,2^, Amare Hailu^3^, Daniel A. Wiegant^3^, Bernice S. Scholten^1^, Paul J. Van den Brink^1,4,*^*

^1^ Department of Aquatic Ecology and Water Quality Management, Wageningen University, Wageningen University and Research centre, P.O. Box 47, 6700 AA Wageningen, The Netherlands

^2^ The College of Natural Sciences, University of Addis Ababa, Arat Kilo campus, Addis Ababa, Ethiopia

^3^ Horn of Africa Regional Environment Centre and Network, Addis Ababa University, P.O. Box 80773, Addis Ababa, Ethiopia

^4^ Alterra, Wageningen University and Research centre, P.O. Box 47, 6700 AA Wageningen, The Netherlands

Table SM1 HC5/HC50 values and the lower and upper limits of HC5 and HC50 values

| **Pesticides** | **#of data points** | **HC5**  **(µg/L)** | **LL HC5**  **(µg/L)** | **ULHC5**  **(µg/L)** | **HC50**  **(µg/L)** | **LLHC50**  **(µg/L)** | **ULHC50**  **(µg/L)** |
| --- | --- | --- | --- | --- | --- | --- | --- |
| Sulphur | 7 | 6.90E+02 | 2.91E+00 | 9.87E+00 | 2.02E+05 | 1.83E+04 | 2.24E+06 |
| Endosulfan | 37 | 4.83E-02 | 1.15E-02 | 1.15E-01 | 6.25E+00 | 2.77E+00 | 1.41E+00 |
| Diazinon | 34 | 5.06E-01 | 1.88E-01 | 1.07E+00 | 1.23E+01 | 7.04E+00 | 2.15E+01 |
| Spiroxamine | 8 | 1.80E-01 | 6.97E-04 | 3.19E+00 | 1.19E+02 | 9.50E+00 | 1.50E+00 |
| Teflubenzuron | 6 | 4.69E-01 | 1.08E-03 | 7.10E+00 | 1.07E+02 | 8.35E+00 | 1.38E+03 |
| Methomyl | 21 | 1.06E+01 | 3.69E+00 | 2.19E+01 | 1.29E+02 | 7.34E+01 | 2.27E+02 |
| Trifloxystrobin | 12 | 1.27E+00 | 2.24E+01 | 3.62E+00 | 2.12E+01 | 8.94E+00 | 5.01E+01 |
| Carbendazim | 11 | 1.91E+01 | 1.45E+00 | 8.63E+01 | 9.52E+02 | 2.70E+02 | 3.36E+03 |
| Deltamethrin | 12 | 2.43E-03 | 2.73E-04 | 9.07E-03 | 8.39E-02 | 2.83E-02 | 2.48E-01 |
| Pyraclostrobin | 14 | 3.49E-01 | 2.89E-02 | 1.67E+00 | 3.13E+01 | 8.85E+00 | 1.11E+02 |

Note: All data passed the Anderson-Darling test at the 0.01 significance level.

Table SM2 Measured pesticide concentrations and associated toxicity values, PNEC and ETR values for all meaured pesticides for each sampling point and year (all in μg/L).

| Compound | Year | Concen-tration | Fish 96hLC50 | PNECfish | Invertebrates 48hEC50 | PNEC invertebrates | Algae 72hEC50 | PNEC Algae | PNEC ug/l | ETR |
| --- | --- | --- | --- | --- | --- | --- | --- | --- | --- | --- |
| **Meki and Ketar** |  |  |  |  |  |  |  |  |  |  |
| Dimethoate | 2010 | 0.03 | 30.2 | 302 | 2 | 20 | 90.4 | 9040 | 20 | 0.0035 |
| Fenitrothion | 2010 | 0.08 | 1.3 | 13 | 0.0086 | 0.086 | 1.3 | 130 | 0.086 | 0.9302326 |
| Metalaxyl | 2010 | 0.11 | 100 | 1000 | 28 | 280 | 33 | 3300 | 280 | 0.2107143 |
| metsulfuron-methyl | 2010 | 0.04 | 150 | 1500 | 150 | 1500 | 0.045 | 4.5 | 4.5 | 0.0088889 |
| piperonyl butoxide | 2010 | 0.02 | 5.3 | 53 | 0.51 | 5.1 | 0.24 | 24 | 5.1 | 0.0039216 |
| Sulphur | 2010 | 7 | 0.063 | 0.63 | 0.063 | 0.63 | 0.063 | 6.3 | 0.63 | 11.111111 |
| Triadimefon | 2010 | 0.04 | 4.08 | 40.8 | 7.16 | 71.6 | 2.01 | 201 | 40.8 | 0.0009804 |
| Antraquinone | 2014 | 0.11 | 72 | 720 | 10 | 100 | 10 | 1000 | 100 | 0.0011 |
| Difenyl(Bifenyl) | 2014 | 0.013 | 1.5 | 15 | 2.38 | 23.8 | 1.3 | 130 | 15 | 0.0008667 |
| Endosulfan | 2014 | 0.139 | 0.01 | 0.1 | 0.76 | 7.6 | 2.15 | 215 | 0.1 | 1.39 |
| Imidacloprid | 2014 | 0.014 | 83 | 830 | 85 | 850 | 10 | 1000 | 830 | 0.0002289 |
| Boscalid | 2015 | 0.1 | 2.7 | 27 | 5.33 | 53.3 | 3.75 | 375 | 27 | 0.0037037 |
| Carbendazim | 2015 | 0.82 | 0.19 | 1.9 | 0.15 | 1.5 | 7.7 | 770 | 1.5 | 0.5466667 |
| Dimethoate | 2015 | 0.07 | 30.2 | 302 | 2 | 20 | 90.4 | 9040 | 20 | 0.0035 |
| Imidacloprid | 2015 | 0.19 | 83 | 830 | 85 | 850 | 10 | 1000 | 830 | 0.0002289 |
| Metalaxyl | 2015 | 59 | 100 | 1000 | 28 | 280 | 33 | 3300 | 280 | 0.2107143 |
| Methoxyfenozide | 2015 | 0.056 | 4.2 | 42 | 3.7 | 37 | 3.4 | 340 | 37 | 0.0015135 |
| Prochloraz | 2015 | 0.15 | 1.5 | 15 | 4.3 | 43 | 0.0055 | 0.55 | 0.55 | 0.2727273 |
| Thiacloprid | 2015 | 0.098 | 24.5 | 245 | 85.1 | 851 | 60.6 | 6060 | 245 | 0.0004 |
| Triadimenol | 2015 | 0.059 | 21.3 | 213 | 51 | 510 | 9.6 | 960 | 213 | 0.000277 |
| **Kontola and Gura** | |  |  |  |  |  |  |  |  |  |
| Carbaryl | 2010 | 0.05 | 2.6 | 26 | 0.0064 | 0.064 | 0.6 | 60 | 0.064 | 0.78125 |
| Diazinon | 2010 | 0.09 | 3.1 | 31 | 0.01 | 0.1 | 6.4 | 640 | 0.1 | 2.8 |
| Hexaflumuron | 2010 | 0.01 | 100 | 1000 | 0.0001 | 0.001 | 3.2 | 320 | 0.001 | 10 |
| Linuron | 2010 | 0.02 | 3.15 | 31.5 | 0.31 | 3.1 | 0.016 | 1.6 | 1.6 | 0.0125 |
| Metalaxyl | 2010 | 0.09 | 100 | 1000 | 28 | 280 | 33 | 3300 | 280 | 0.0003214 |
| Met-sulfuron-methyl | 2010 | 0.08 | 100 | 1000 | 100 | 1000 | 0.2 | 20 | 20 | 0.004 |
| Sulphur | 2010 | 3 | 0.063 | 0.63 | 0.063 | 0.63 | 0.063 | 6.3 | 0.63 | 4.7619048 |
| Thiamethoxam | 2010 | 0.01 | 125 | 1250 | 100 | 1000 | 100 | 10000 | 1000 | 0.000079 |
| Triadimefol | 2010 | 0.02 | NA | NA | NA | NA | NA | NA | NA | NA |
| Actamiprid | 2014 | 0.08 | 100 | 1000 | 49.8 | 498 | 98.3 | 9830 | 498 | 0.0001606 |
| Antraquinone | 2014 | 0.018 | 72 | 720 | 10 | 100 | 10 | 1000 | 100 | 0.00018 |
| Bitertanol | 2014 | 0.54 | 2.14 | 21.4 | 4.46 | 44.6 | 1.38 | 138 | 21.4 | 0.0252336 |
| Bosicalid | 2014 | 11 | 2.7 | 27 | 5.33 | 53.3 | 3.75 | 37.5 | 27 | 0.4074074 |
| Buprofezin | 2014 | 0.88 | 0.33 | 3.3 | 0.42 | 4.2 | 2.1 | 210 | 3.3 | 0.2666667 |
| Carbendazim | 2014 | 0.02 | 0.19 | 1.9 | 0.15 | 1.5 | 7.7 | 770 | 1.5 | 0.0133333 |
| Chlorantraniliprole | 2014 | 0.013 | 12 | 120 | 0.0116 | 0.116 | 4 | 400 | 0.116 | 0.112069 |
| Cyprodinil | 2014 | 0.11 | 2.41 | 24.1 | 0.22 | 2.2 | 2.6 | 260 | 2.2 | 0.05 |
| Diazinon | 2014 | 0.28 | 3.1 | 31 | 0.01 | 0.1 | 6.4 | 640 | 0.1 | 2.8 |
| Dodemorph | 2014 | 32 | 2.2 | 22 | 3.34 | 33.4 | 17.9 | 1790 | 22 | 1.4545455 |
| Fenithrothion | 2014 | 0.78 | 0.23 | 2.3 | 0.4 | 4 | 0.024 | 2.4 | 2.3 | 0.3391304 |
| Hexythiazox | 2014 | 0.029 | 3.2 | 32 | 0.47 | 4.7 | 0.4 | 40 | 4.7 | 0.0061702 |
| Kresoxim-methyl | 2014 | 1.4 | 0.19 | 1.9 | 0.19 | 1.9 | 0.06 | 6 | 1.9 | 0.7368421 |
| Lufenuron | 2014 | 0.08 | 29 | 290 | 0.0013 | 0.013 | 8.8 | 880 | 0.013 | 6.1538462 |
| Metalaxyl | 2014 | 0.011 | 100 | 1000 | 28 | 280 | 33 | 3300 | 280 | 0.0003214 |
| Propoxur | 2014 | 0.026 | 6.2 | 62 | 0.15 | 1.5 | NA | NA | 1.5 | 0.0173333 |
| Pyrimethanil | 2014 | 0.016 | 10.56 | 105.6 | 2.9 | 29 | 1.2 | 120 | 29 | 0.0005517 |
| Spiroxamine | 2014 | 57 | 7.13 | 71.3 | 6.1 | 61 | 0.003 | 0.3 | 0.3 | 190 |
| Teflubenzuron | 2014 | 0.032 | 0.0065 | 0.065 | 0.0028 | 0.028 | 0.02 | 2 | 0.028 | 1.1428571 |
| Thiamethoxam | 2014 | 0.079 | 125 | 1250 | 100 | 1000 | 100 | 10000 | 1000 | 0.000079 |
| Triadimefol | 2014 | 0.037 | NA | NA | NA | NA | NA | NA | NA | NA |
| Bosicalid | 2015 | 0.21 | 2.7 | 27 | 5.33 | 53.3 | 3.75 | 37.5 | 27 | 0.4074074 |
| Buprofezin | 2015 | 0.24 | 0.33 | 3.3 | 0.42 | 4.2 | 2.1 | 210 | 3.3 | 0.2666667 |
| Dodemorph | 2015 | 0.48 | 2.2 | 22 | 3.34 | 33.4 | 17.9 | 1790 | 22 | 1.4545455 |
| **Floriculture** |  |  |  |  |  |  |  |  |  |  |
| Acetamiprid | 2009 | 7.6 | 100 | 1000 | 49.8 | 498 | 98.3 | 9830 | 498 | 0.015261 |
| azoxystrobin | 2009 | 2.2 | 0.47 | 4.7 | 0.23 | 2.3 | 0.36 | 36 | 2.3 | 0.9565217 |
| Bitertanol | 2009 | 0.11 | 2.14 | 21.4 | 4.46 | 44.6 | 1.38 | 138 | 21.4 | 0.0373832 |
| Carbendazim | 2009 | 9.1 | 0.19 | 1.9 | 0.15 | 1.5 | 7.7 | 770 | 1.5 | 6.0666667 |
| Cyprodinil | 2009 | 0.19 | 2.41 | 24.1 | 0.22 | 2.2 | 2.6 | 260 | 2.2 | 0.0863636 |
| Diethyltoluamide | 2009 | 0.14 | 71.3 | 713 | 75 | 750 | NA | NA | 713 | 0.0001964 |
| Dimethomorph | 2009 | 0.77 | 3.4 | 34 | 10.6 | 106 | 29.2 | 2920 | 34 | 0.0226471 |
| Dodemorph | 2009 | 0.5 | 2.2 | 22 | 3.34 | 33.4 | 17.9 | 1790 | 22 | 0.0227273 |
| Ethirimol | 2009 | 0.01 | 60.8 | 608 | 50 | 500 | 24 | 2400 | 500 | 0.0024 |
| Fenamiphos-sulfoxide | 2009 | 0.02 | 100 | 1000 | 0.015 | 0.15 | NA | NA | NA | NA |
| Fludioxonil | 2009 | 0.26 | 0.23 | 2.3 | 0.27 | 2.7 | 0.024 | 2.4 | 2.3 | 0.1130435 |
| Imidacloprid | 2009 | 0.15 | 83 | 830 | 85 | 850 | 10 | 1000 | 830 | 0.0003614 |
| Iprodione | 2009 | 0.81 | 3.7 | 37 | 0.66 | 6.6 | 1.8 | 180 | 6.6 | 0.1227273 |
| Iprovalicarb | 2009 | 0.04 | 22.7 | 227 | 19.8 | 198 | 10 | 1000 | 198 | 0.0019192 |
| Linuron | 2009 | 0.02 | 3.15 | 31.5 | 0.31 | 3.1 | 0.016 | 1.6 | 1.6 | 0.0125 |
| Metalaxyl | 2009 | 0.51 | 100 | 1000 | 28 | 280 | 33 | 3300 | 280 | 0.0018214 |
| Methomyl | 2009 | 2.7 | 0.63 | 6.3 | 0.0076 | 0.076 | 100 | 10000 | 0.076 | 35.526316 |
| Methoxyfenozide | 2009 | 0.01 | 4.2 | 42 | 3.7 | 37 | 3.4 | 340 | 37 | 0.0135135 |
| Propamocarb | 2009 | 1 | 96.8 | 968 | 106 | 1060 | 301 | 30100 | 968 | 0.0010331 |
| Pyraclostrobin | 2009 | 0.03 | 0.006 | 0.06 | 0.016 | 0.16 | 0.843 | 84.3 | 0.06 | 2.5 |
| Spiroxamine | 2009 | 4 | 7.13 | 71.3 | 6.1 | 61 | 0.003 | 0.3 | 0.3 | 13.333333 |
| Tebufenpyrad | 2009 | 0.11 |  | 0 |  | 0 |  | 0 | 0 | NA |
| Tetradifon | 2009 | 1.15 | 880 | 8800 | 2 | 20 | 100 | 10000 | 20 | 0.0575 |
| Triforine | 2009 | 0.4 | 1000 | 10000 | 25 | 250 | 380 | 38000 | 250 | 0.0016 |
| Bitertanol | 2010 | 0.8 | 2.14 | 21.4 | 4.46 | 44.6 | 1.38 | 138 | 21.4 | 0.0373832 |
| Boscalid | 2010 | 2.6 | 2.7 | 27 | 5.33 | 53.3 | 3.75 | 375 | 27 | 0.4814815 |
| Bupirimate | 2010 | 0.19 | 1 | 10 | 3.41 | 34.1 | 1.6 | 160 | 10 | 0.042 |
| Carbendazim | 2010 | 0.5 | 0.19 | 1.9 | 0.15 | 1.5 | 7.7 | 770 | 1.5 | 6.0666667 |
| Clofentezine | 2010 | 0.1 | 0.015 | 0.15 | 0.0008 | 0.008 | 0.32 | 32 | 0.008 | 12.5 |
| Cyprodinil | 2010 | 0.05 | 2.41 | 24.1 | 0.22 | 2.2 | 2.6 | 260 | 2.2 | 0.0863636 |
| Diethyltoluamide | 2010 | 0.06 | 71.3 | 713 | 75 | 750 | NA | NA | 713 | 0.0001964 |
| Dimethomorph | 2010 | 0.09 | 3.4 | 34 | 10.6 | 106 | 29.2 | 2920 | 34 | 0.0226471 |
| Dodemorph | 2010 | 0.13 | 2.2 | 22 | 3.34 | 33.4 | 17.9 | 1790 | 22 | 0.0227273 |
| Endosulfan | 2010 | 0.06 | 0.01 | 0.1 | 0.76 | 7.6 | 2.15 | 215 | 0.1 | 0.6 |
| Ethirimol | 2010 | 0.32 | 60.8 | 608 | 50 | 500 | 24 | 2400 | 500 | 0.0024 |
| Fenamidone | 2010 | 0.08 | 0.74 | 7.4 | 0.19 | 1.9 | 3.84 | 384 | 1.9 | 0.0421053 |
| Fenamiphos-sulfone | 2010 | 0.01 | NA | NA | NA | NA | NA | NA | NA | NA |
| Fenamiphos-sulfoxide | 2010 | 0.07 | 100 | 1000 | 0.015 | 0.15 | NA | NA | NA | NA |
| Fenarimol | 2010 | 0.4 | 4.1 | 41 | 6.8 | 68 | 1.48 | 148 | 41 | 0.0097561 |
| Fenhexamid | 2010 | 0.08 | 1.34 | 13.4 | 18.8 | 188 | 26.1 | 2610 | 13.4 | 0.0059701 |
| Fenithrothion | 2010 | 0.16 | 0.23 | 2.3 | 0.4 | 4 | 0.024 | 2.4 | 2.3 | 0.0695652 |
| Hexythiazox | 2010 | 0.09 | 3.2 | 32 | 0.47 | 4.7 | 0.4 | 40 | 4.7 | 0.0191489 |
| Imidacloprid | 2010 | 0.3 | 83 | 830 | 85 | 850 | 10 | 1000 | 830 | 0.0003614 |
| Iprovalicarb | 2010 | 0.38 | 22.7 | 227 | 19.8 | 198 | 10 | 1000 | 198 | 0.0019192 |
| Metalaxyl | 2010 | 0.18 | 100 | 1000 | 28 | 280 | 33 | 3300 | 280 | 0.0018214 |
| Methiocarb | 2010 | 0.04 | 0.65 | 6.5 | 0.008 | 0.08 | 2.2 | 220 | 0.08 | 0.5 |
| Methoxyfenozide | 2010 | 0.5 | 4.2 | 42 | 3.7 | 37 | 3.4 | 340 | 37 | 0.0135135 |
| Oxamyl | 2010 | 0.01 | 3.13 | 31.3 | 0.319 | 3.19 | 0.93 | 93 | 3.19 | 0.0031348 |
| Piperonyl-butoxide | 2010 | 0.02 | 5.3 | 53 | 0.51 | 5.1 | 0.24 | 24 | 5.1 | 0.0039216 |
| Propamocarb | 2010 | 0.38 | 96.8 | 968 | 106 | 1060 | 301 | 30100 | 968 | 0.0010331 |
| Pyraclostrobin | 2010 | 0.15 | 0.006 | 0.06 | 0.016 | 0.16 | 0.843 | 84.3 | 0.06 | 2.5 |
| Tetradifon | 2010 | 0.4 | 880 | 8800 | 2 | 20 | 100 | 10000 | 20 | 0.0575 |
| thiophanate-methyl | 2010 | 0.05 | 11 | 110 | 5.4 | 54 | 25.4 | 2540 | 54 | 0.0009259 |
| Triadimefol | 2010 | 0.1 | NA | NA | NA | NA | NA | NA | NA | NA |
| Triadimefon | 2010 | 0.16 | 4.08 | 40.8 | 7.16 | 71.6 | 2.01 | 201 | 40.8 | 0.0039216 |
| Trifloxystrobin | 2010 | 0.34 | 0.015 | 0.15 | 0.11 | 1.1 | 0.0053 | 0.53 | 0.15 | 2.2666667 |
| Triforine | 2010 | 0.1 | 1000 | 10000 | 25 | 250 | 380 | 38000 | 250 | 0.0016 |
| Acetamiprid | 2014 | 2 | 100 | 1000 | 49.8 | 498 | 98.3 | 9830 | 498 | 0.015261 |
| Antraquinone | 2014 | 0.011 | 72 | 720 | 10 | 100 | 10 | 1000 | 100 | 0.00011 |
| Bitertanol | 2014 | 0.49 | 2.14 | 21.4 | 4.46 | 44.6 | 1.38 | 138 | 21.4 | 0.0373832 |
| Boscalid | 2014 | 13 | 2.7 | 27 | 5.33 | 53.3 | 3.75 | 375 | 27 | 0.4814815 |
| Buprofezin | 2014 | 0.12 | 0.33 | 3.3 | 0.42 | 4.2 | 2.1 | 210 | 3.3 | 0.0363636 |
| Chlorantraniliprole | 2014 | 0.027 | 12 | 120 | 0.0116 | 0.116 | 4 | 400 | 0.116 | 0.2327586 |
| Cyprodinil | 2014 | 0.074 | 2.41 | 24.1 | 0.22 | 2.2 | 2.6 | 260 | 2.2 | 0.0863636 |
| Diazinon | 2014 | 0.01 | 3.1 | 31 | 0.01 | 0.1 | 6.4 | 640 | 0.1 | 0.1 |
| Difenyl(Bifenyl) | 2014 | 0.01 | 1.5 | 15 | 2.38 | 23.8 | 1.3 | 130 | 15 | 0.0006667 |
| Difenylamine | 2014 | 0.033 | 2.2 | 22 | 1.2 | 12 | 0.3 | 30 | 12 | 0.00275 |
| Dimethomorph | 2014 | 0.49 | 3.4 | 34 | 10.6 | 106 | 29.2 | 2920 | 34 | 0.0226471 |
| Dodemorph | 2014 | 0.014 | 2.2 | 22 | 3.34 | 33.4 | 17.9 | 1790 | 22 | 0.0227273 |
| Fenamidone | 2014 | 0.025 | 0.74 | 7.4 | 0.19 | 1.9 | 3.84 | 384 | 1.9 | 0.0421053 |
| Fipronil | 2014 | 0.011 | 0.248 | 2.48 | 0.19 | 1.9 | 0.068 | 6.8 | 1.9 | 0.0057895 |
| Fludioxonil | 2014 | 0.2 | 0.23 | 2.3 | 0.27 | 2.7 | 0.024 | 2.4 | 2.3 | 0.1130435 |
| Fluopyram | 2014 | 0.039 | 0.98 | 9.8 | 100 | 1000 | 1.13 | 113 | 9.8 | 0.0056122 |
| Flusilazole | 2014 | 0.019 | 1.2 | 12 | 3.4 | 34 | 6.4 | 640 | 12 | 0.0015833 |
| Imidacloprid | 2014 | 0.028 | 83 | 830 | 85 | 850 | 10 | 1000 | 830 | 0.0003614 |
| Kerosoxim-methyl | 2014 | 0.047 | 0.19 | 1.9 | 0.19 | 1.9 | 0.06 | 6 | 1.9 | 0.0247368 |
| Mandipropamid | 2014 | 0.2 | 2.9 | 29 | 7.1 | 71 | 19.8 | 1980 | 29 | 0.0068966 |
| Metalaxyl | 2014 | 0.086 | 100 | 1000 | 28 | 280 | 33 | 3300 | 280 | 0.0018214 |
| Methoxyfenozide | 2014 | 0.16 | 4.2 | 42 | 3.7 | 37 | 3.4 | 340 | 37 | 0.0135135 |
| penconazole | 2014 | 0.02 | 1.13 | 11.3 | 6.75 | 67.5 | 4.9 | 490 | 11.3 | 0.0115044 |
| Propoxur | 2014 | 0.053 | 6.2 | 62 | 0.15 | 1.5 | NA | NA | 1.5 | 0.0353333 |
| Pyraclostrobin | 2014 | 0.02 | 0.006 | 0.06 | 0.016 | 0.16 | 0.843 | 84.3 | 0.06 | 2.5 |
| Pyrimethanil | 2014 | 0.019 | 10.56 | 105.6 | 2.9 | 29 | 1.2 | 120 | 29 | 0.0006552 |
| Spiroxamine | 2014 | 0.015 | 7.13 | 71.3 | 6.1 | 61 | 0.003 | 0.3 | 0.3 | 13.333333 |
| Teflubenzuron | 2014 | 0.049 | 0.0065 | 0.065 | 0.0028 | 0.028 | 0.02 | 2 | 0.028 | 1.75 |
| tetraconazole | 2014 | 0.027 | 4.3 | 43 | 3 | 30 | 2.4 | 240 | 30 | 0.0009 |
| Thiacloprid | 2014 | 0.14 | 24.5 | 245 | 85.1 | 851 | 60.6 | 6060 | 245 | 0.0005714 |
| Thiamethoxam | 2014 | 0.53 | 125 | 1250 | 100 | 1000 | 100 | 10000 | 1000 | 0.00053 |
| Acetamiprid | 2015 | 0.9 | 100 | 1000 | 49.8 | 498 | 98.3 | 9830 | 498 | 0.015261 |
| Boscalid | 2015 | 5.4 | 2.7 | 27 | 5.33 | 53.3 | 3.75 | 375 | 27 | 0.4814815 |
| Bupirimate | 2015 | 0.42 | 1 | 10 | 3.41 | 34.1 | 1.6 | 160 | 10 | 0.042 |
| Buprofezin | 2015 | 0.12 | 0.33 | 3.3 | 0.42 | 4.2 | 2.1 | 210 | 3.3 | 0.0363636 |
| Dichlooraniline (3,5-) | 2015 | 0.16 | 1.94 | 19.4 | 0.12 | 1.2 | 1.65 | 165 | 1.2 | 0.1333333 |
| Dimethomorph | 2015 | 0.11 | 3.4 | 34 | 10.6 | 106 | 29.2 | 2920 | 34 | 0.0226471 |
| Ethirimol | 2015 | 1.2 | 60.8 | 608 | 50 | 500 | 24 | 2400 | 500 | 0.0024 |
| Fludioxonil | 2015 | 0.13 | 0.23 | 2.3 | 0.27 | 2.7 | 0.024 | 2.4 | 2.3 | 0.1130435 |
| Fluopyram | 2015 | 0.055 | 0.98 | 9.8 | 100 | 1000 | 1.13 | 113 | 9.8 | 0.0056122 |
| Imidacloprid | 2015 | 0.055 | 83 | 830 | 85 | 850 | 10 | 1000 | 830 | 0.0003614 |
| Mandipropamid | 2015 | 0.19 | 2.9 | 29 | 7.1 | 71 | 19.8 | 1980 | 29 | 0.0068966 |
| Methoxyfenozide | 2015 | 0.059 | 4.2 | 42 | 3.7 | 37 | 3.4 | 340 | 37 | 0.0135135 |
| penconazole | 2015 | 0.13 | 1.13 | 11.3 | 6.75 | 67.5 | 4.9 | 490 | 11.3 | 0.0115044 |
| **Water supply treatment plant** | | |  |  |  |  |  |  |  |  |
| Methomyl | 2009 | 0.02 | 0.63 | 6.3 | 0.0076 | 0.076 | 100 | 10000 | 0.076 | 0.2631579 |
| Pyridate metabolite (CHPP) | 2009 | 0.01 | 1.01 | 10.1 | 0.49 | 4.9 | 0.75 | 75 | 4.9 | 0.0020408 |
| Isoproturon | 2010 | 0.03 | 32 | 320 | 16 | 160 | 0.052 | 5.2 | 5.2 | 0.0057692 |
| Metsulfuron-methyl | 2010 | 0.3 | 100 | 1000 | 100 | 1000 | 0.2 | 20 | 20 | 0.015 |
| Sulphur | 2010 | 10 | 0.063 | 0.63 | 0.063 | 0.63 | 0.063 | 6.3 | 0.63 | 15.873016 |
| Buprofezin | 2014 | 0.036 | 0.33 | 3.3 | 0.42 | 4.2 | 2.1 | 210 | 3.3 | 0.0212121 |
| Deet | 2014 | 0.014 | NA | NA | NA | NA | NA | NA | 0 | NA |
| Deltamethrin | 2014 | 0.01 | 0.00026 | 0.0026 | 0.00056 | 0.0056 | 9.1 | 910 | 0.0026 | 3.8461538 |
| Diazinon | 2014 | 0.41 | 3.1 | 31 | 0.01 | 0.1 | 6.4 | 640 | 0.1 | 4.1 |
| Dodemorph | 2014 | 0.017 | 2.2 | 22 | 3.34 | 33.4 | 17.9 | 1790 | 22 | 0.0007727 |
| Endosulfan | 2014 | 0.103 | 0.01 | 0.1 | 0.76 | 7.6 | 2.15 | 215 | 0.1 | 1.03 |
| Fenhexamid | 2014 | 0.043 | 1.34 | 13.4 | 18.8 | 188 | 26.1 | 2610 | 13.4 | 0.003209 |
| Lufenuron | 2014 | 0.018 | 29 | 290 | 0.0013 | 0.013 | 8.8 | 880 | 0.013 | 1.3846154 |
| Teflubenzuron | 2014 | 0.08 | 0.0065 | 0.065 | 0.0028 | 0.028 | 0.02 | 2 | 0.028 | 2.8571429 |
| Buprofezin | 2015 | 0.07 | 0.33 | 3.3 | 0.42 | 4.2 | 2.1 | 210 | 3.3 | 0.0212121 |
| Pyraclostrobin | 2015 | 0.062 | 0.006 | 0.06 | 0.016 | 0.16 | 0.843 | 84.3 | 0.06 | 1.0333333 |
| **Bulbula** |  |  |  |  |  |  |  |  |  |  |
| Antraquinone | 2014 | 0.027 | 72 | 720 | 10 | 100 | 10 | 1000 | 100 | 0.00027 |
| Biteranol | 2014 | 0.034 | 2.14 | 21.4 | 4.46 | 44.6 | 1.38 | 138 | 21.4 | 0.0015888 |
| Buprofezin | 2014 | 0.081 | 0.33 | 3.3 | 0.42 | 4.2 | 2.1 | 210 | 3.3 | 0.0245455 |
| Cyprodinil | 2014 | 0.021 | 2.41 | 24.1 | 0.22 | 2.2 | 2.6 | 260 | 2.2 | 0.0095455 |
| Diazinon | 2014 | 0.043 | 3.1 | 31 | 0.01 | 0.1 | 6.4 | 640 | 0.1 | 0.43 |
| Difenyl(Bifenyl) | 2014 | 0.018 | 1.5 | 15 | 2.38 | 23.8 | 1.3 | 130 | 15 | 0.0012 |
| Difenylamine | 2014 | 0.022 | 2.2 | 22 | 1.2 | 12 | 0.3 | 30 | 12 | 0.0018333 |
| Dodemorph | 2014 | 0.42 | 2.2 | 22 | 3.34 | 33.4 | 17.9 | 1790 | 22 | 0.0190909 |
| Kresoxim-methyl | 2014 | 0.018 | 0.19 | 1.9 | 0.19 | 1.9 | 0.06 | 6 | 1.9 | 0.0094737 |
| Spiroxamine | 2014 | 6.9 | 7.13 | 71.3 | 6.1 | 61 | 0.003 | 0.3 | 0.3 | 23 |
| Teflubenzuron | 2014 | 0.014 | 0.0065 | 0.065 | 0.0028 | 0.028 | 0.02 | 2 | 0.028 | 0.5 |
| Boscalid | 2015 | 0.17 | 2.7 | 27 | 5.33 | 53.3 | 3.75 | 375 | 27 | 0.0062963 |
| dodemorph | 2015 | 0.052 | 2.2 | 22 | 3.34 | 33.4 | 17.9 | 1790 | 22 | 0.0023636 |
